# Supplementary material for: Spatial Variation in Stream-Water Bacterial Communities Across Sites Differing in Wild Chinese Giant Salamander Detection Frequency
Source: Biology (Basel). 2026 Sep 18;15(18):1647. doi: 10.3390/biology15181647 (PMC13604818; doi:10.3390/biology15181647)
Supplement: Supplementary file 1 [file biology-15-01647-s001.zip › Data Related to “Spatial Variation in Stream-Water Bacterial Communities across Sites Differing in Wild Chinese Giant Salamander Detection Frequency”/Microbial Data/result/03.TaxonVisual/ternary/sample/class/S1-S3-S6.ternary.pdf]

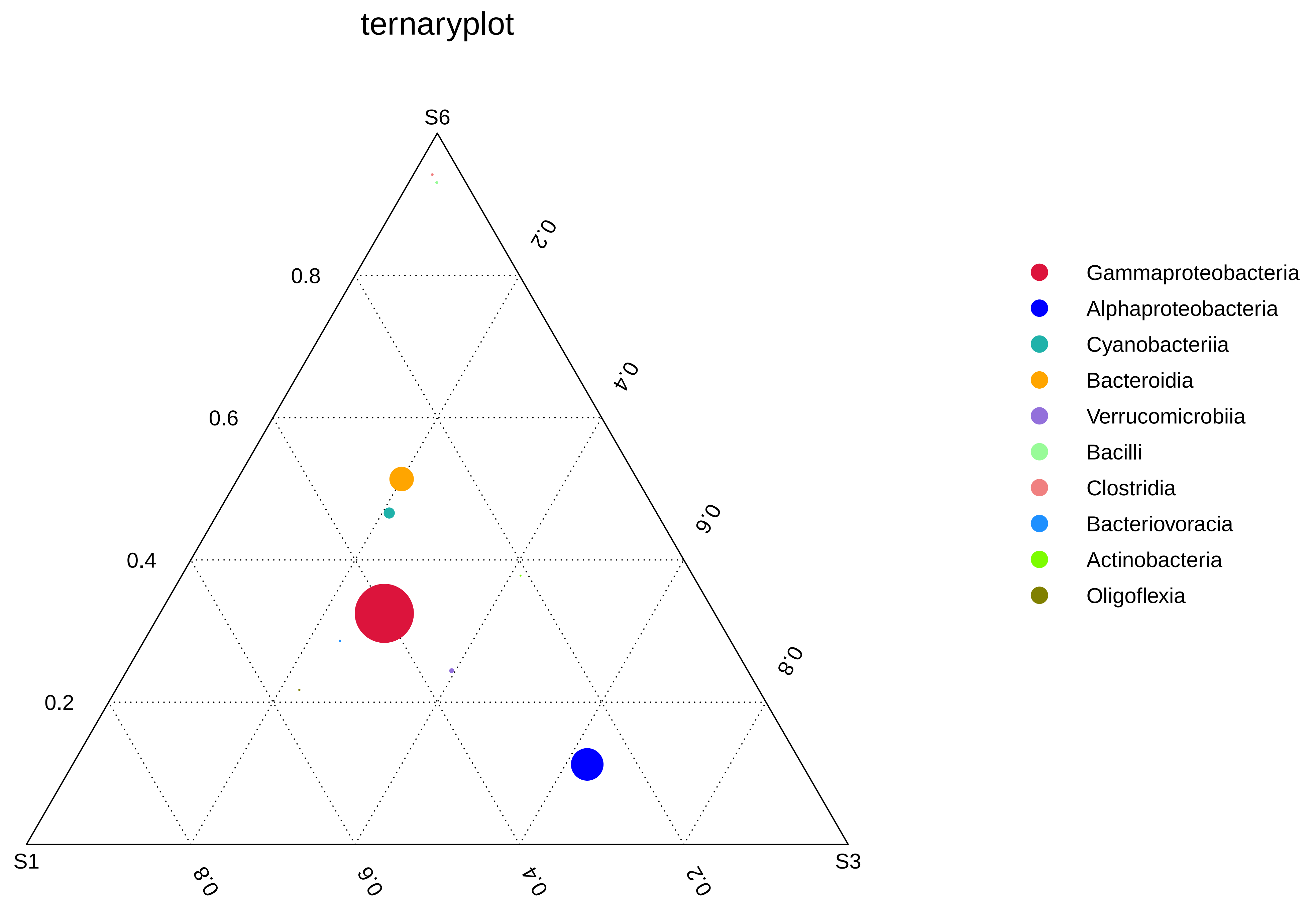
ternaryplot
S6
S1
S3
0.2
0.4
0.6
0.8
Gammaproteobacteria
Alphaproteobacteria
Cyanobacteriia
Bacteroidia
Verrucomicrobiia
Bacilli
Clostridia
Bacteriovoracia
Actinobacteria
Oligoflexia
